# An Unconcealed Reservoir: Case Report of Erosion of Inflatable Penile Implant Reservoir through the Anus

**DOI:** 10.1155/2020/8850974

**Published:** 2020-10-05

**Authors:** Mark Arthur, Jonathan Canete, Charles Welliver

**Affiliations:** ^1^Albany Medical College, Department of General Surgery, Division of Urology, Albany, NY, USA; ^2^Albany Medical College, Department of General Surgery, Section of Colon and Rectal Surgery, Albany, NY, USA; ^3^Albany Stratton Veterans Affairs Medical Center, Albany, NY, USA

## Abstract

Inflatable penile prosthesis (IPP) involves placement of the fluid reservoir into an abdominal or pelvic location. While the space of Retzius (SOR) was the preferred site for many years, the change from open to robotic prostatectomy made this space less desirable due to the violation of the peritoneum with the robotic approach. Other factors like previous abdominal or pelvic surgeries (particularly inguinal hernia repair with mesh) may also require a change in location of the reservoir during IPP placement. In this report, we discuss a previously undescribed result of alternative reservoir placement (ARP) with erosion of the reservoir through the colon and out of the anus in a man with multiple previous abdominal surgeries. Management of this clinical problem is also discussed.

## 1. Introduction

Inflatable penile prosthetics (IPP) are a surgical treatment of erectile dysfunction (ED) often reserved for medically refractory cases. The typical IPP consists of 3 parts: inflatable cylinders within the penis, a scrotal pump, and a fluid reservoir which is placed in the abdomen or pelvis.

Traditionally, the fluid reservoir was placed within the space of Retzius (SOR) for IPP. With the change to robotically assisted laparoscopic prostatectomy (RALP) and resulting concerns about the safety of the SOR after the violation of the peritoneum that occurs with RALP, alternative reservoir placement (ARP) sites have grown in popularity [1]. The abdominal wall space avoids the SOR but has its own unique set of complications [2–4].

In this case, we present a unique device herniation and resulting patient management. In this case, a patient's reservoir eroded through the colon and ultimately out of his anus.

## 2. Case Presentation

**Figure 1:**
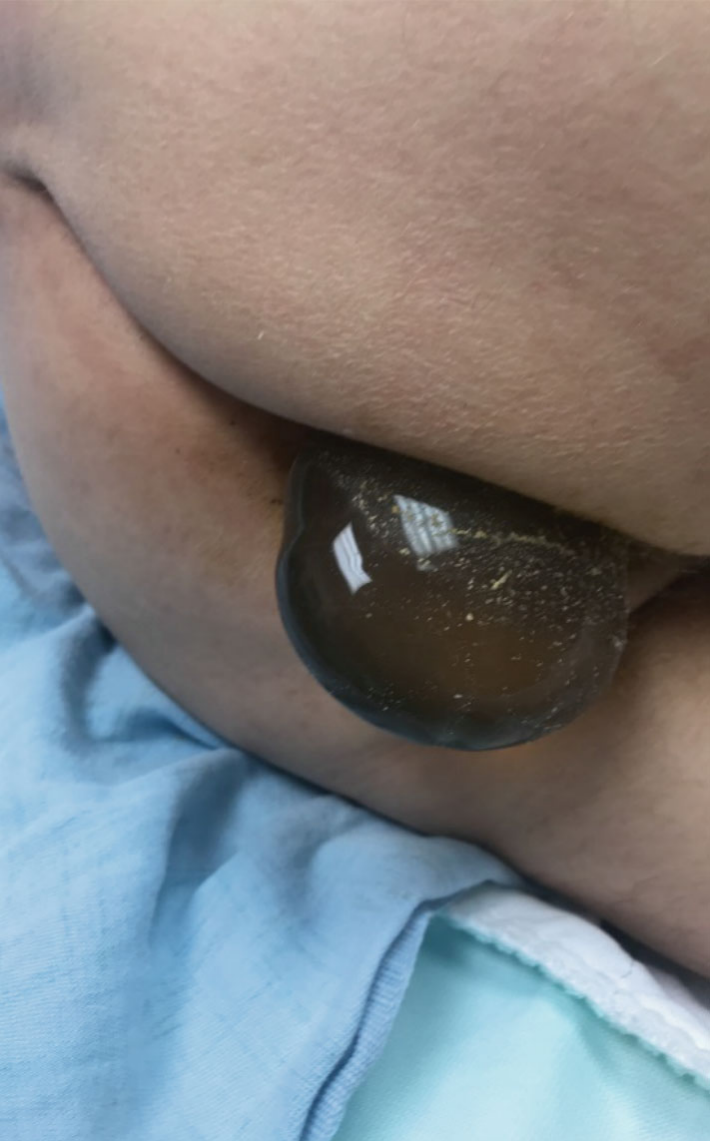
Posterior view of reservoir protrusion through the anus.

**Figure 2:**
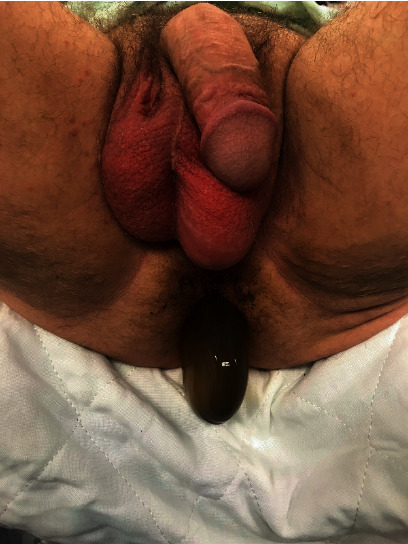
Inferior view of reservoir protrusion through the anus.

A 56-year-old male patient presented to the emergency department after passing two dark brown, loose stools followed by the feeling of something protruding from his anus. The protrusion was observed to be the reservoir for his IPP (Figures 1 and 2).

The patient's past medical history includes an IPP (AMS CX 700, Boston Scientific, Marlborough, Massachusetts) placed through a penoscrotal approach three years prior to presentation at our emergency department. The reservoir was placed via abdominal incision due to previous bilateral inguinal hernia repair with mesh. The reservoir was placed below the rectus muscles on the right side after entering through the fascia. A month prior to his presentation at our institution, he had a laparoscopic appendectomy complicated with postoperative ileus. At the time of clinical presentation in the emergency department, his white blood cell count was not elevated and he was afebrile. His scrotum was mildly erythematous, but otherwise, his IPP was not concerning for clinical infection.

**Figure 3:**
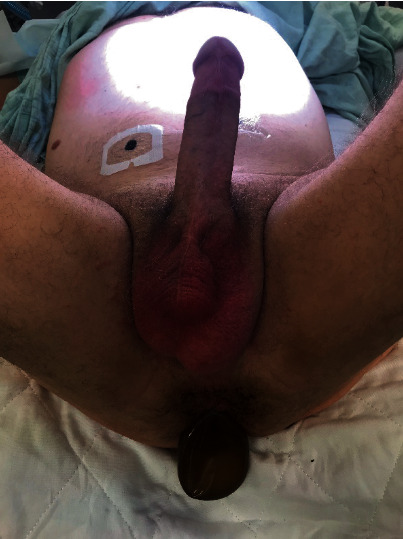
Protrusion of a deflated reservoir through the anus demonstrating maintained function of implant device.

He was initially treated with intravenous antibiotics and scheduled for surgery the next day. His IPP was found to still be functional before surgery (Figure 3). During surgery, urology removed the IPP and colorectal surgery performed a diagnostic laparoscopy that did not identify an intraperitoneal component of the penile implant. Loop sigmoid colostomy was then created. Anorectal examination using an anoscope could not identify laceration or other injury to the colon even to a distance 10 cm proximal to the anal verge. After IPP removal and washout, a malleable implant was placed through the penoscrotal incision.

At urology follow-up, the malleable implant was without signs of infection. At six-week surgical follow-up with colorectal surgery, a gastrografin enema was performed to evaluate the integrity of the rectum, and a small linear streak of contrast was seen emanating from the anterior aspect of the rectum. Spontaneous resolution was anticipated, and the patient was advised to keep the loop colostomy. At the next follow-up two months later, another gastrografin enema was performed and leakage from the rectum was not observed at which point the colostomy was reversed, and normal bowel function resumed.

## 3. Discussion

Historically, placement of the reservoir during IPP was through the inguinal canal into the SOR. However, ARP is gaining popularity as this approach does not risk damaging retropubic structures and can be done safely in patients with prior pelvic surgeries (principally RALP) [1, 2]. In this case, ARP was required as the SOR was inaccessible due to previous bilateral hernia repair with mesh. Unfortunately, ARP and the subsequent surgeries led to a previously undescribed finding of the reservoir protruding from the anus.

The advent and resulting increase in the use of RALP, with its violation of the peritoneum to access the pelvis, changed how implanters approach placement of the reservoir. ARP does allow for implants to avoid the SOR but is not without its own unique set of complications. As most penile implants are placed via penoscrotal incision [5], the placement into either the SOR or the ARP is done blindly. In an interesting study looking at reservoir insertion through the penoscrotal approach into a projected high submuscular space, 80% were found in the ideal spot anterior to the transversalis muscle. However, reservoirs were also found in the retroperitoneal (10%), preperitoneal (5%), and intraperitoneal spaces (5%) [6]. In a multicenter study looking at the need for surgical revisions of IPP reservoirs, statistically similar rates were seen between ARP and SOR placement (2.0 vs 1.3%, *p* = 0.44) with reservoir fluid leakage the most common reason for device revision [2].

While our complication of the reservoir protruding from the anus has not been previously described, erosion into visceral strictures including the bowel is not unique. A comprehensive review and case series done by Levine et al. in 2012 found 8 cases with reservoir herniation into the bowel, 6 into the small intestine, and 2 into the large intestine [7]. All 8 individuals had prior pelvic or abdominal surgeries. Other older reports have also described bowel erosion even during the time of standard SOR reservoir placement [8, 9].

The nonemergent way this clinical problem was managed is also notable. While the implant was obviously infected due to the bowel-related erosion and exposure of the reservoir, the patient presented without life threatening or urgent signs of infection. In this case, intravenous antibiotics were started to decrease any surrounding soft tissue infection around the device with surgical treatment the next day. Malleable implant was then placed which has a high success rate even with clinically infected implants [10].

## 4. Conclusion

Previous abdominal surgery is clearly a risk factor for visceral erosion of IPP reservoirs but may be managed with antibiotics and delayed malleable implant placement in patients without concerning clinical signs of infection.

## Data Availability

Patient-specific data is not publicly available due to privacy restrictions. All other data analyzed is listed in the references and may be accessed through PubMed.
